# Bayesian Inference Underlies the Contraction Bias in Delayed Comparison Tasks

**DOI:** 10.1371/journal.pone.0019551

**Published:** 2011-05-12

**Authors:** Paymon Ashourian, Yonatan Loewenstein

**Affiliations:** 1 Department of Brain and Cognitive Sciences, Massachusetts Institute of Technology, Cambridge, Massachusetts, United States of America; 2 Department of Neurobiology, Edmond and Lily Safra Center for Brain Sciences, The Interdisciplinary Center for Neural Computation and Center for the Study of Rationality, The Hebrew University of Jerusalem, Jerusalem, Israel; Monash University, Australia

## Abstract

Delayed comparison tasks are widely used in the study of working memory and perception in psychology and neuroscience. It has long been known, however, that decisions in these tasks are biased. When the two stimuli in a delayed comparison trial are small in magnitude, subjects tend to report that the first stimulus is larger than the second stimulus. In contrast, subjects tend to report that the second stimulus is larger than the first when the stimuli are relatively large. Here we study the computational principles underlying this bias, also known as the contraction bias. We propose that the contraction bias results from a Bayesian computation in which a noisy representation of a magnitude is combined with a-priori information about the distribution of magnitudes to optimize performance. We test our hypothesis on choice behavior in a visual delayed comparison experiment by studying the effect of (i) changing the prior distribution and (ii) changing the uncertainty in the memorized stimulus. We show that choice behavior in both manipulations is consistent with the performance of an observer who uses a Bayesian inference in order to improve performance. Moreover, our results suggest that the contraction bias arises during memory retrieval/decision making and not during memory encoding. These results support the notion that the contraction bias illusion can be understood as resulting from optimality considerations.

## Introduction

Comparing magnitudes of two temporally separated stimuli is one of the fundamental tools of experimental psychology and neuroscience. Interestingly, choice behavior in these experiments reveals a fundamental bias: when the first stimulus is small, subjects tend to overestimate it, whereas when it is large, they tend to underestimate it. The first account of this bias, known as the contraction bias, was published a century ago by Harry Levi Hollingworth who later became one of the pioneers of applied psychology. Hollingsworth presented subjects with square cards of various sizes for a brief period of time and asked them to memorize their sizes [1]. Each card presentation was followed by a short delay, after which the subjects selected a matching card from a set of probe cards. Surprisingly, Hollingsworth observed that subjects tended to choose a probe card that was too large when the memorized card was small compared to the other cards used in the experiment, whereas the opposite behavior, i.e. picking too small a probe card, was observed when the memorized card was relatively large. This illusion has been demonstrated numerous times since Hollingworth's publication for a variety of analog magnitudes in the visual, auditory, and somatosensory modalities [1]–[7], [ for review see 8].

The customary explanation for the contraction bias is that the *perceived* magnitude of a stimulus is a weighted combination of its veridical magnitude and a reference magnitude, such as an average of all contextually relevant stimuli, that serves as an anchor [3], [9], [ but see 10]. Thus in Hollingsworth's experiments and others [1]–[5] the anchor is thought to make a larger contribution to the subjective magnitude of the memorized stimulus than to the subjective magnitude of the probe stimulus. As a result, the memorized stimulus is biased towards the anchor more than the probe stimulus, which results in the overestimation of small memorized stimuli and the underestimation of large memorized stimuli. This explanation, however, is at best partial since there is no consensus on the choice of the contextually relevant stimuli that comprise the anchor, or on the relative weights of the physical and reference magnitudes. Moreover, it is not clear why the weight applied to the memorized stimulus should be different from the weight applied to the probe stimulus. Finally, the computational principles underlying this bias remain unknown. In order to address these questions we explored whether the contraction bias can be understood as resulting from optimality considerations.

There is a growing body of literature suggesting that the brain utilizes Bayes' rule to optimally combine information from different sources [11]–[18]. In particular, the application of Bayes' rule has been demonstrated in slant perception [19], sensorimotor learning [20], speed estimation [18], time estimation and interval timing [21], motion perception [22], and integration of information from different sensory modalities [12], [23]. In addition, it has been suggested that Bayesian inference underlies the effect of categories on behavior in reconstruction tasks [24]. Therefore, we hypothesized that the contraction bias in delayed comparison tasks results from a Bayesian inference in which noisy representations of stimuli are combined with knowledge about the a-priori distribution of magnitudes in order to optimize performance. Intuitively, such an inference should lead to the contraction bias because the perception of extreme magnitudes of the first stimulus, which are unlikely given unimodal prior distributions, will be biased toward the ‘center’ of the prior distribution.

In order to test this hypothesis, we conducted an experiment in which we instructed subjects to memorize the length of a bar presented on a computer screen and then compare this memorized length to the length of a probe bar. We show that contraction bias depends on the prior distribution of bar lengths, and increasing the uncertainty in the memory of bar lengths enhances the contraction bias, both of which are consistent with the Bayesian hypothesis.

When within a trial does the Bayesian computation take place? Is the encoded memory biased or does the prior information bias the result of the length comparison? By manipulating uncertainty in the memory of bar lengths *after* memory encoding and measuring the magnitude of the contraction bias we demonstrate that prior information is introduced during memory retrieval/decision making rather than when the first stimulus is encoded in memory.

Some of the findings presented here have appeared previously in abstract form [25].

## Results

### Example of Contraction Bias

**Figure 1:**
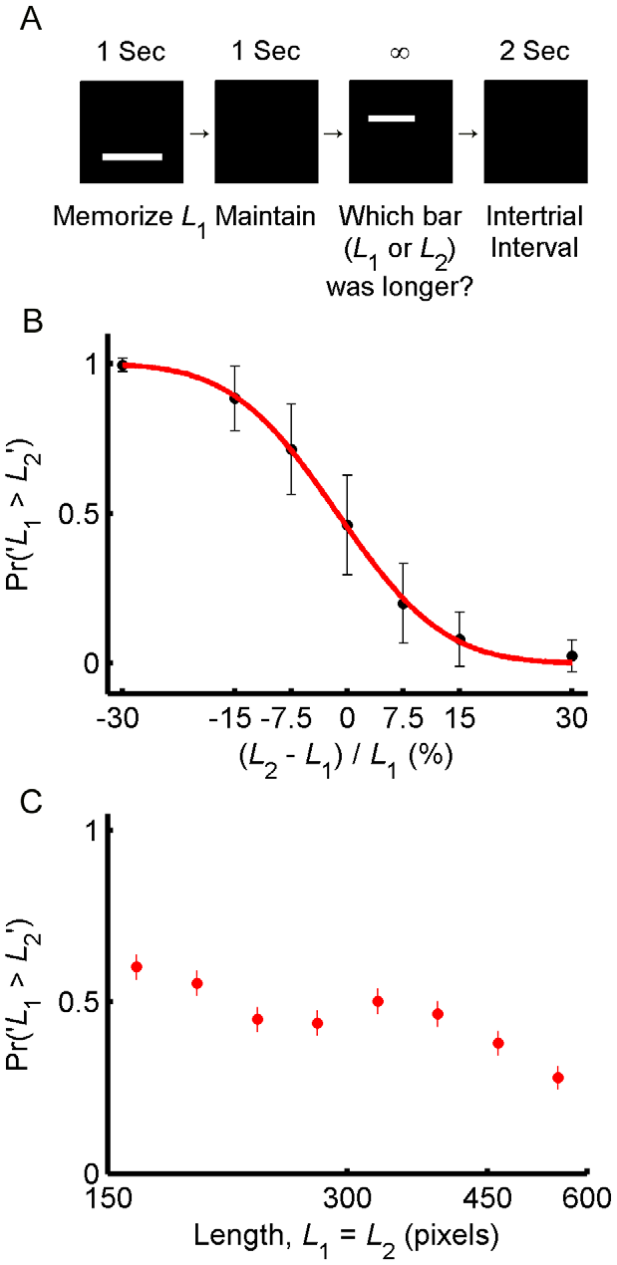
The delayed comparison task and subjects' performance. **A**, The standard task. Subjects viewed a horizontal bar (*L*_1_) on a computer screen for 1 sec and memorized its length. After a delay period of 1 sec, during which the screen remained blank, the subjects viewed a second bar (*L*_2_) and were instructed to report which of the two bars was longer. The second bar, *L*_2_ remained visible until subjects made a response. The difference in length between *L_1_* and *L_2_* varied between −30% and +30%. Unbeknownst to the subjects, on roughly 50% of the trials, the lengths of the first and second bars were equal (*L*_1_ = *L*_2_). **B**, The average psychometric curve of 9 subjects. The abscissa corresponds to the difference between the two bar lengths, 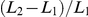 and the ordinate corresponds to the fraction of trials in which subjects chose *L*_1_ as longer than *L*_2_. Error bars depict standard error of the mean (SEM). Line is a least-square fit of an error function: 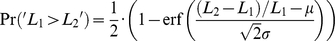 where 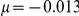 and 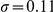. **C**, Average response curve of 9 subjects. Fraction of times in which subjects reported ‘*L*_1_>*L*_2_’ on the impossible trials are plotted as a function of bar length. Subjects overestimated the magnitude of the memorized *L*_1_ bar when it was relatively small and underestimated *L*_1_ when it was relatively long, consistent with the contraction bias. Each data point corresponds to 21 impossible trials per subject. Error bars depict SEM.

In the standard task (Figure 1A), subjects viewed a horizontal bar (*L_1_*) for 1 sec and were instructed to memorize its length. After a delay of 1 sec, during which screen remained blank, they viewed a probe bar (*L_2_*). The probe bar remained visible on the screen until subjects reported which of the two bars was longer by pressing dedicated keys on the keyboard. The first bar, *L*_1_, was drawn from a uniform distribution in the logarithmic scale between 150 and 600 pixels. The difference in length between *L_1_* and *L_2_* varied between −30% and +30%. Both bars were presented at random locations on the screen and no feedback was provided to the subjects on performance on individual trials (See Materials and Methods). We quantified the proficiency of individual subjects on the delayed comparison task by measuring psychometric curves that depict the percentage of ‘*L*_1_>*L*_2_’ responses as a function of the difference between the memorized and probe stimuli. The average psychometric curve of the subjects (*n* = 9) is plotted in Figure 1B, showing that accuracy improved as the absolute difference between the lengths of *L*_1_ and *L*_2_ increased.

Our purpose is to quantify the contraction bias in these experiments. Previous studies have demonstrated a contraction bias in delayed comparison tasks by showing that the *pattern* of errors made by subjects depends on the magnitude of the memorized stimulus. When the memorized stimulus is small, subjects tend to make more errors in trials in which the probe stimulus is larger than the memorized stimulus, compared to trials in which the probe is smaller than the memorized stimulus. The opposite behavior is observed when the magnitude of the memorized stimulus is large [1]–[5]. However, these errors only provide a qualitative measure of the bias because the number of errors depends on the relative difficulty of the task, i.e., the difference between the two stimuli (*L*_1_ and *L*_2_ in our experiments) relative to the width of the psychometric curve. We used a different approach to overcome this limitation: Unbeknownst to the subjects, we included a subset of trials in which the lengths of the two bars were identical. We term these trials “impossible trials” because there is no correct answer to the question “which bar (*L*_1_ or *L*_2_) was longer”. Impossible trials are well suited for the analysis of the contraction bias because performance on these trials is independent of the proficiency of individual subjects in distinguishing the difference in the length of the two bars.

The average response curve of 9 subjects is depicted in Figure 1C, where we plot the percentage of trials in which the subjects reported that ‘*L*_1_>*L*_2_’ as a function of the length of *L*_1_ (*L*_1_ = *L*_2_). Note that despite the fact that *L*_1_ and *L*_2_ were identical on these trials, subjects reported that *L*_1_ was longer than *L*_2_ on roughly 60% of the shortest trials (left-most point in Figure 1C) whereas they reported *L*_1_ was longer than *L*_2_ only in 28% of the longest trials (right-most point in Figure 1C). The slope of the regression line fitted to the impossible trials was significantly smaller than zero (mean slope = −0.28, 95% bootstrap confidence interval (CI) = [−0.36, −0.21], see Materials and Methods for procedure), indicating that subjects were more likely to report ‘*L*_1_>*L*_2_’ for shorter *L*_1_ bars as compared to longer *L*_1_ bars, thus exhibiting the contraction bias.

### Bayesian Inference and Contraction Bias

**Figure 2:**
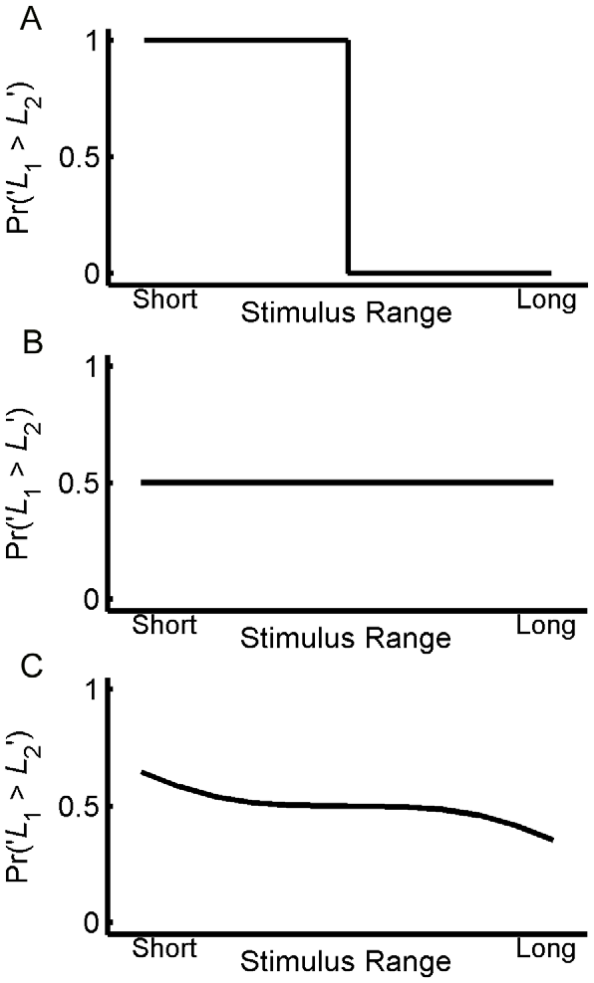
Bayesian inference and the contraction bias. **A**, Response curve of a Bayesian model with infinite noise in the representation of *L*_1_ (no memory of *L*_1_) and no noise in representation of *L*_2_. The model reports ‘*L*_1_>*L*_2_’ in trials where *L*_2_ is smaller than the median of the prior distribution, and ‘*L*_1_<*L*_2_’ in trials in which *L*_2_ is larger than the median. This behavior arises because the posterior distribution of *L*_1_ is the same as the prior distribution of the bar lengths, whereas the posterior distribution of *L*_2_ is not influenced by the prior at all. **B**, Response curve of the Bayesian model with equal noise in the representations of *L*_1_ and of *L*_2_. The contribution of the prior to the posteriors of *L*_1_ and *L*_2_ is identical because the levels of noise in the two representations are equal. Thus, in trials where *L*_1_ = *L*_2_, the model reports ‘*L*_1_>*L*_2_’ at chance level independently of the length of the bars. **C**, Response curve of the Bayesian model assuming that there is more noise in the representation of *L*_1_ than in representation of *L*_2_. Response curve is a combination of **A** and **B**.

**Figure 3:**
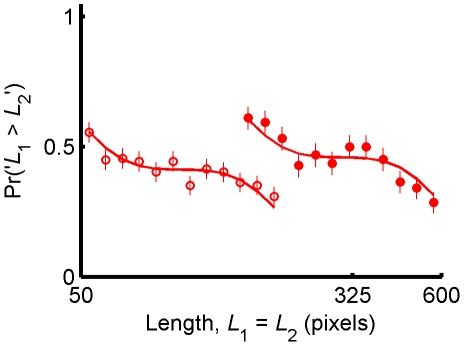
Effect of the prior on the response curve. Assuming that noise is independent of bar length, the model predicts that the shape of the response curve is independent of the physical range of the stimuli. Thus, a lateral shift in the prior would result in a lateral shift in the response curve. Two groups of subjects completed the task in Figure 1A for two overlapping uniform priors, 50 to 200 pixels (open circles) and 150 to 600 pixels (filled circles). The response curve in the impossible trials was not significantly different between the two groups. Each data point corresponds to 14 impossible trials per subject. Error bars depict SEM. Lines are the best fit of the Bayesian model, see Materials and Methods.

Our aim is to account for the contraction bias in a Bayesian framework of decision making. In order to see how the contraction bias emerges from Bayesian inference, we consider a control region in the brain, such as the prefrontal cortex [26], that is presented with the *neural representations* of *L*_1_ and *L*_2_ and has to decide which of the two bars is longer. We assume that: (1) the control region knows that the neural representations of *L*_1_ and *L*_2_ are noisy, e.g. due to noise in the sensory pathway. Moreover the representation of *L*_1_ is noisier than that of *L*_2_ because *L*_1_ has to be stored in memory, a process that may contribute additional noise to the representation of *L*_1_; (2) the control region has information about the marginal distribution of bar lengths. This distribution can be approximated based on the history of the experiment; (3) the control region utilizes Bayes' rule and combines the noisy representations of *L*_1_ and *L*_2_ with knowledge about the prior distribution in order to construct the posterior distributions for the two bar lengths. These posteriors are then used to minimize error in judgment. A formal description of this process appears in the Materials and Methods section. To illustrate how the contraction bias could emerge from such a Bayesian computation, we consider the following three examples:

1. ***L*** **_1_ is unknown,** ***L*** **_2_ is known**. Consider a hypothetical subject who forgets the length of *L*_1_, but has no ambiguity about the length of *L*_2_, i.e., the neural representation of *L*_1_ is infinitely noisy whereas there is no noise in the neural representation of *L*_2_. In this case, the posterior of *L*_1_ is the prior distribution. In contrast, the prior distribution makes no contribution to the posterior of *L*_2_. Therefore, the optimal strategy would be to report ‘*L*_1_>*L*_2_’ in trials where *L*_2_ is smaller than the median of the prior distribution and to report ‘*L*_1_<*L*_2_’ in trials in which *L*_2_ is larger than the median. Therefore, in the impossible trials in which *L*_1_ = *L*_2_, the subject would report ‘*L*_1_>*L*_2_’ if *L*_1_ is smaller than the median of the prior distribution and would report ‘*L*_1_<*L*_2_’ otherwise, as depicted in Figure 2A. This response pattern is consistent with the contraction bias because it appears as though the subject is overestimating relatively small *L*_1_ and underestimating relatively large *L*_1_.
2. ***L*** **_1_ and** ***L*** **_2_ are equally uncertain**. Consider a case where the estimated uncertainties in the representations of *L*_1_ and *L*_2_ are equal. This would be true if the only uncertainty in the representations of *L*_1_ and *L*_2_ results from sensory noise, and memory storage does not add any additional noise to the representation of *L*_1_. In the impossible trials in which the two bars are physically identical, the contribution of the prior distribution to the posteriors of *L*_1_ and *L*_2_ is equal. Symmetry considerations indicate that the subject would report that ‘*L*_1_>*L*_2_’ at chance level for all bar lengths, i.e., there is no contraction bias, as depicted in Figure 2B.
3. ***L*** **_1_ is less certain than** ***L*** **_2_**. In intermediate cases where the level of uncertainty in *L*_1_ is larger than that of *L*_2_, for example, as a result of added noise due to memory storage, we expect the resultant response curve to reside between the response curves of Figures 3A and 3B, resulting in a smooth decrease in the fraction of trials in which *L*_1_ is reported to be larger than *L*_2_ as a function of the lengths of *L*_1_ and *L*_2_ (Figure 2C).

### Model Predictions and Behavioral Results

#### Effect of Changing the Prior

If the contraction bias results from Bayesian inference, then changing the prior distribution is expected to change the response curve. In particular, assuming that noise is independent of the length of the bars, a translational shift in the prior distribution would result in an equal translational shift in the response curve without changing its shape. To test this prediction, we asked a new group of naïve subjects to participate in the experiment of Figure 1A, in which *L*_1_ was drawn from a new uniform distribution in the logarithmic scale between 50 and 200 pixels (*n* = 10). Similar to the first experiment, all lengths were presented in logarithmic scale to satisfy the assumption of independence of noise and bar length. We compared the responses of this group to the original group who saw stimuli that were drawn from a uniform distribution in the logarithmic scale between 150 to 600 pixels. The accuracy of subjects in the trials in which *L*_1_≠*L*_2_ (non-impossible trials) was indistinguishable between the two groups (83%±2% for 50–200; 85%±1% for 150–600; *t*_17_ = 0.69, *p* = 0.49, two-tailed), supporting the assumption that the level of noise in the neural representation of the bars is independent of bar length in these ranges. Response curves for the two groups in the impossible trials are depicted in Figure 3 (50–200 pixels: open circles; 150–600 pixels: filled circles). The slope of a regression line fitted to the response curve for the 50–200 pixel group was significantly smaller than zero (mean slope = −0.20, 95% bootstrap CI = [−0.27, −0.13]). Response curve slopes were not significantly different between the group who saw 50–200 pixel lines and the original group who saw 150–600 pixel lines (average difference = −0.08; 95% bootstrap CI = [−0.19 +0.02]). Thus, as predicted by the Bayesian hypothesis, a translational shift in the prior distribution resulted in a translational shift in the response curve.

Qualitatively, the shape of the response curve does not seem linear. Rather, the slope of the curve is more negative for bar lengths at the high and low ends of the spectrum. The non-linear response curve is consistent with our Bayesian model whose two parameters, the level of noise in the representation of the two bars, were chosen to minimize the fit mean square error. The resultant parameters indicate that the uncertainty in the representation of *L*_1_ is 30% higher than the uncertainty in *L*_2_ (Materials and Methods). Moreover, the Bayesian model is qualitatively similar to the experimental results, supporting our hypothesis that the contraction bias results from Bayesian inference.

#### Effect of Noise

According to the Bayesian hypothesis, the contraction bias emerges because the contribution of the prior distribution to the posterior of the first bar is larger than the contribution of prior to the posterior of the second bar. This asymmetry results from the fact that the uncertainty in the representation of the memorized bar, *L*_1_, is larger than that of the probe bar, *L*_2_. The larger the asymmetry in the contribution of the prior to the posteriors of the two bars, the more pronounced the contraction bias should be. Therefore, increasing the level of noise in the representation of *L*_1_ is expected to enhance this asymmetry and thus enhance the contraction bias.

**Figure 4:**
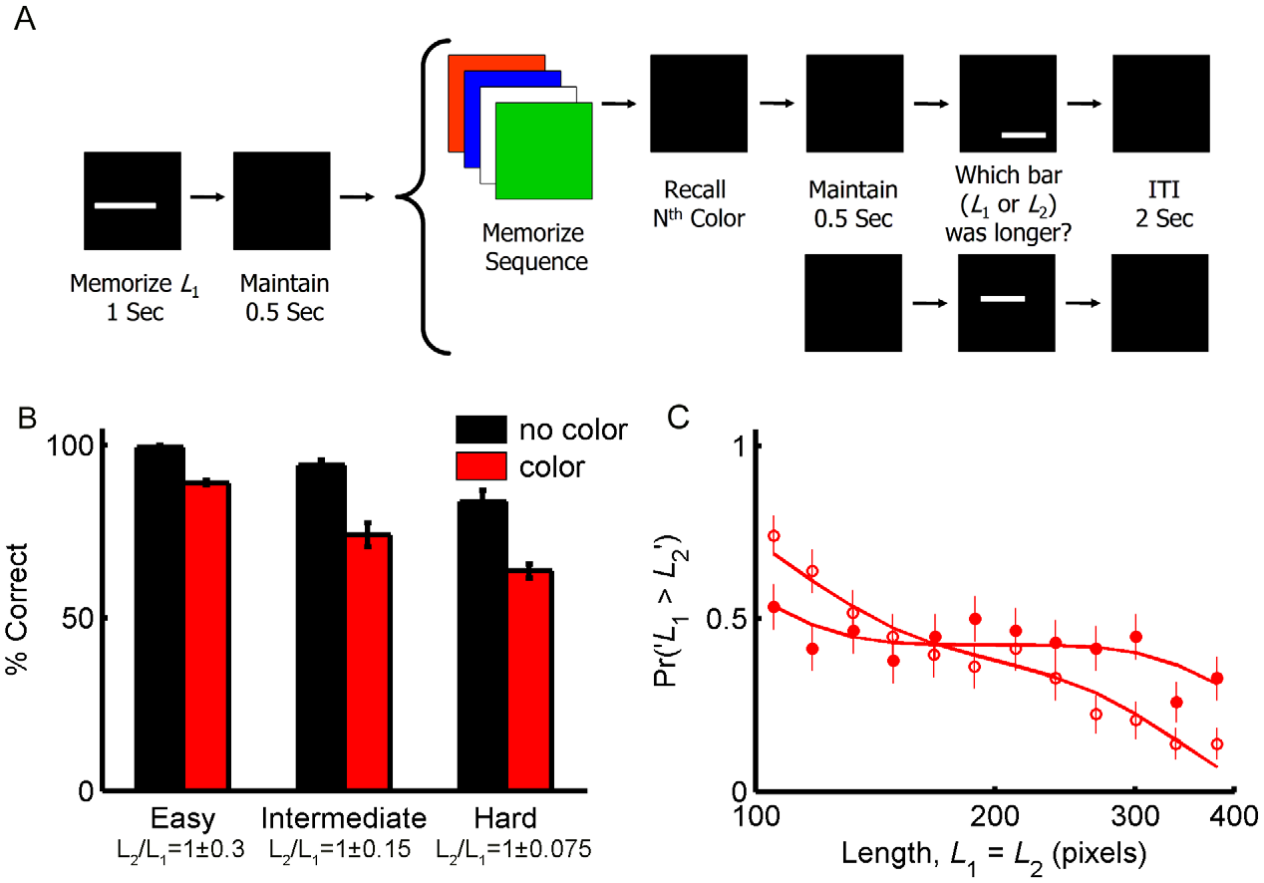
Effect of noise on the response curve. **A**, Subjects performed a modified experiment where a secondary task had to be performed between the presentations of the two bars on randomly selected 50% of the trials. Top row depicts sequence of events in trials with interference: a sequence of 4 colors was presented on the screen 500 msec after the presentation of *L*_1_. Each color was presented for 400 msec and subjects were instructed to memorize the sequence. 400 msec after the disappearance of the last color, a number from 1 to 4 appeared on the screen. Subjects were instructed to recall the color that corresponded to the number. **B**, Percentage correct in bar length comparison in the standard (black) and modified (red) trials. The ability to memorize the length of *L*_1_ was impaired in the modified trials compared to the standard unperturbed trials, in both the easy (±30%, left), intermediate (±15%, center) and hard (±7.5%, right) trials. These results suggest that the secondary task increased uncertainty in the memory of the length of *L*_1_. **C**, Response curve in the standard (filled circles) and modified trials (open circles). The larger slope of the response curve on the modified trials compared to the standard trials suggests that the secondary task caused an enhancement of the contraction bias. Each data point corresponds to 6 impossible trials per subject. Error bars depict SEM. Lines are the best fit of the Bayesian model, see Materials and Methods.

To test this prediction, we modified the task of Figure 1A and added a distracting memory task between the presentations of the two bars in randomly selected half of the trials: 500 msec after the disappearance of *L*_1_, four different colors were flashed on the screen in a random order for 400 msec each (Figure 4A). Then, subjects were instructed to recall the *n*'th presented color, where *n* was a number between 1 and 4, appearing on the screen immediately after the last color. Following the answer, *L*_2_ was presented and subjects were instructed to compare it with *L*_1_ as before. On average, subjects correctly recalled the color in 96% of the trials indicating that the color task was not disregarded.

The distracting task was designed to interfere with the memory of the first bar. In the Bayesian framework, it was intended to add ‘noise’ to the representation of *L*_1_. As predicted, accuracy of performance on the bar comparison task, measured in the trials in which *L*_1_≠*L*_2_, was lower on the trials interrupted by the secondary task compared to the performance on trials that were not interrupted by the secondary task (Figure 4B). This decrease in performance was significant both in the easiest trials in which the difference between *L*_1_ and *L*_2_ was ±30% (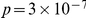, two-tailed *t*-test), the intermediate trials in which the difference between *L*_1_ and *L*_2_ was ±15% (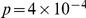, two-tailed *t*-test) and the most difficult trials in which the difference between *L*_1_ and *L*_2_ was ±7.5% (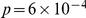, two-tailed *t*-test).

In order to characterize the effect of the secondary task on the contraction bias, we compared the response curve of subjects in the impossible trials with interference from the secondary task (open circles in Figure 4C) with the response curve of the same subjects in the impossible trials with no interference from the secondary task (filled circles in Figure 4C). The slope of the linear fit to the response curve in trials devoid of the secondary task was −0.19 (95% bootstrap CI = [−0.32, −0.07]). In contrast, the slope of the linear fit to the response curves in trials with the secondary task was −0.63 (95% bootstrap CI = [−0.75, −0.53]), which was significantly more negative than the slope in trials without the secondary task (average difference = −0.45; 95% bootstrap CI = [−0.61 −0.28]). These results suggest that, as predicted by the Bayesian model, an increase in internal noise, which manifests as a decrease in behavioral accuracy, is associated with an increase in the level of contraction bias, which manifests as an increase in the magnitude of the slope of the response curve. To further test this hypothesis, we examined the accuracy of performance on an individual basis by fitting a psychometric curve (cumulative Gaussian function similar to Figure 1B ) to the responses of each subject, once in trials with and then in trials without the distracting task, and estimating the width of the psychometric curve (σ) in each case. Next, we calculated the correlation between the slope of the linear fit to the response curve of each subject (i.e. the magnitude of contraction bias) and their respective psychometric σ. The correlation coefficient between values of σ and the slopes of the response curves was −0.74 (*p* = 0.0002, two-tailed), supporting the assertion that a decrease in performance is associated with an increase in the magnitude of the contraction bias.

## Discussion

We examined the hypothesis that the contraction bias in delayed comparison tasks results from a Bayesian inference in which information about the prior distribution is combined with noisy measurement in order to optimize performance. This hypothesis makes two predictions: a translational shift in the prior distribution is expected to result in a similar translational shift in the bias curve, and increasing noise in memory is expected to increase reliance on prior knowledge and thus increase the bias. Our results are consistent with both predictions, suggesting that the contraction bias results from a Bayesian inference.

Within a single trial, when does information about the prior distribution combine with the sensory measurement? One possibility is that it takes place during the encoding of *L*_1_. In this case, the encoded memory of *L*_1_ is already biased in the direction of the prior distribution. Another possibility is that the memory of *L*_1_ is unbiased and the Bayesian computation takes place at the comparison stage, when the encoded *L*_1_ is compared with *L*_2_. To address this question we again considered the choice behavior of subjects in the experiment with the interfering task. We found that in this experiment, the slope of the response curve was more negative in trials with interference from the secondary task, compared to the standard trials (Figure 4C). In other words, more weight was given to the prior distribution in trials interrupted by the secondary task. Recall that trials containing this task were randomly intermixed with trials that did not contain interference. Therefore, at the time of encoding of *L*_1_ (up to 0.5 sec after the end of the presentation of *L*_1_) the subjects could not know whether they would be presented with an interfering task and therefore could not know what weight to give to the prior distribution. Therefore, if the computation had taken place at the time of the encoding of *L*_1_, we would have observed no difference in the slope of the response curve between the two conditions. Therefore, the Bayesian computation necessarily took place *after* the interfering task, at the time of *L*_1_ retrieval or later, when *L*_1_ and *L*_2_ were compared.

How do subjects learn the prior distribution? In order to address this question, we compared the level of contraction bias, as measured by the slope of the response curve, in the first 20 impossible trials to the slope in the last 20 impossible trials for subjects who completed the experiment in Figure 1A where the bar lengths were drawn from the 150–600 and 50–200 ranges. We found no statistical difference in these slopes (−0.29 for the first 20 trials; −0.28 for the last 20 trials; average difference = −0.01; 95% bootstrap CI for the difference in slopes, [−0.29 0.27]). These results indicate that the contraction bias emerges within a small number of trials, suggesting that the prior distribution of bar lengths in the experiment is estimated using a small number of trials.

In this study we examined the effect of a translational shift in the prior, but we did not alter the shape of the prior distribution. Previous studies have shown that subjects are sensitive to the shape of the prior distribution in category and sensimotor learning [20], [24]. Consistent with these results, changing the shape of the prior distribution in our model changes the shape of the response curve. The extent to which the shape of the prior distribution can be learned and utilized in Bayesian reasoning, however, awaits future studies.

Contraction bias in delayed comparison tasks is a common cognitive illusion observed in many different modalities and under different experimental conditions [1]–[8]. In this paper we provide a normative interpretation of this bias, supported by an experiment in visual domain. Our results are consistent with a growing body of literature showing that the brain utilizes close-to-optimal computational strategies.

## Materials and Methods

### Ethics Statement

All subjects gave written informed consent using methods approved by the Massachusetts Institute of Technology Committee on the Use of Humans as Experimental Subjects.

### Subjects

Subjects were undergraduate and graduate students from the Massachusetts Institute of Technology. All subjects had normal or corrected-to-normal vision and no subjects took part in more than one of the experiments. Each subject received $10 plus 1 cent for every correct trial in the experiment for a session lasting less than an hour.

### Stimuli

Stimuli were white horizontal bars on a black background displayed on a 17″ computer screen with a resolution of 1024×768. All bars were 3 pixels wide.

### Procedure

Subjects sat approximately 60 cm from a computer screen in a dimly lit room. Each subject completed 400 to 600 trials in one hour and received feedback on their overall performance after every 20 trials. No other feedback was provided. In the standard task, each trial started with the presentation of a *L*_1_ at a random location on the screen for 1 sec. After a delay period of 1 sec, during which screen remained blank, *L*_2_ appeared at another random location on the screen. *L*_2_ remained visible until the subjects pressed one of two keys indicating which bar was longer. The difference in length between *L_1_* and *L_2_* varied between −30% and +30%. Unbeknownst to the subjects, in roughly 50% of the trials, the lengths of the first and second bars were equal (*L*_1_ = *L*_2_). Subjects did not receive feedback on performance on individual trials. Each trial was followed by a 2 sec intertrial interval during which the screen remained blank. Two distinct groups of subjects completed the standard task. One group (*n* = 9) saw *L*_1_ bars chosen uniformly in the logarithmic scale from the [50, 200] pixel interval, while the other group (*n* = 10) saw bars chosen from the [150, 600] pixel interval.

The modified task was identical to the standard task with two exceptions: (1) *L*_1_ bars were chosen uniformly in the logarithmic scale from the [100, 400] pixel interval; (2) subjects completed a distracting memory task between the presentation of *L*_1_ and *L*_2_ in a randomly selected 50% of the trials: 500 msec after *L*_1_ disappeared, a random sequence of four colors (red, blue, white, and green) were displayed on the screen for 400 msec each. 400 msec after the disappearance of the last color, a number from 1 to 4 appeared in yellow on the screen. Subjects were instructed to recall the color that corresponded to the number and press one of four dedicated keys to indicate this color. *L*_2_ appeared 500 msec after subjects made their color choice.

### A Bayesian Model of Contraction Bias

According to our Bayesian hypothesis, the contraction bias emerges because subjects use Bayes' law to combine noisy information about the lengths of the bars with knowledge about the prior information in order to optimize performance. In this section we formalize this intuition.

**Figure 5:**
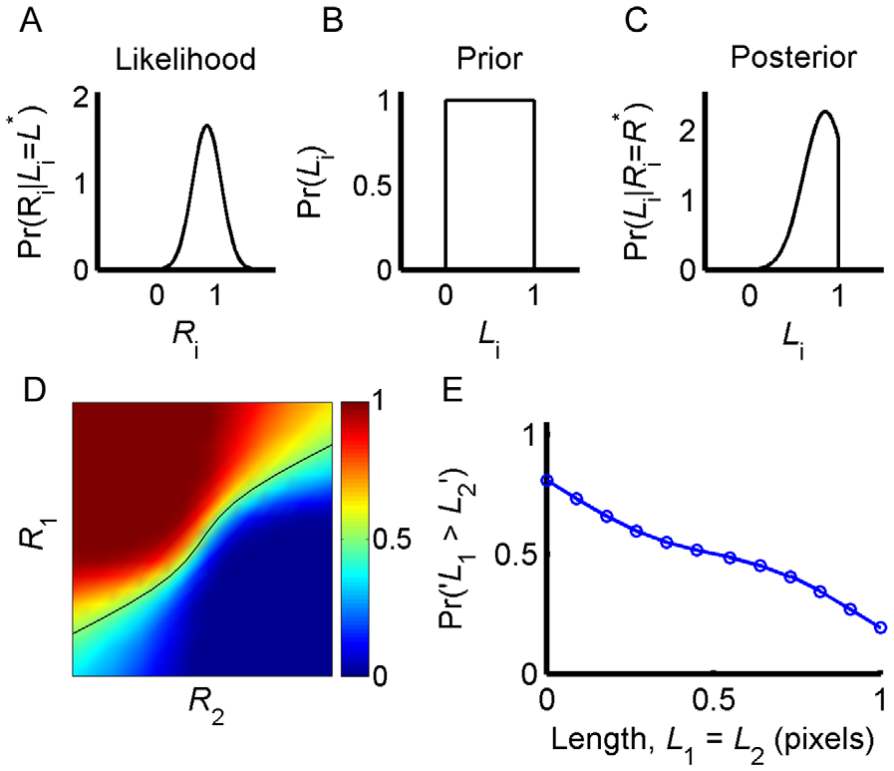
Ideal decision maker solution to the task in **Figure 1A**. **A**, The likelihood of a representation *R_i_* given a particular length (here *L_i_* = 0.85, *σ_i_* = 0.24) assuming 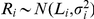. **B**, The prior distribution of bar lengths. **C**, The posterior distribution of *L_i_* given a particular measurement (here *R_i_* = 0.85), calculated using Bayes' rule. **D**, The probability that *L*_1_>*L*_2_ for different values of *R*_1_ and *R*_2_, computed using the posteriors. The black line corresponds to the values of *R*_1_ and *R*_2_ such that Pr(*L*_1_>*L*_2_|*R*_1_,*R*_2_) = 0.5 (here, 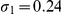 and 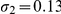). **E**, Response curve of the model on the impossible trials in which *L*_1_ = *L*_2_.

In accordance with Weber's law, the lengths of the bars are measured in logarithmic scale. Let *L_i_* and *R_i_* be the logarithm of the length of bar *i* and its neural representation, respectively. We assume that this representation is noisy such that 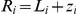 where *z_i_* is drawn from a zero-mean Gaussian distribution with variance 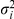, 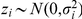. This is illustrated in Figure 5A where we plot the probability of a neural representation *R_i_* for a given representation of bar length 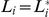, also known as a likelihood function and denoted as 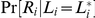. We assume that the prior distribution of bar lengths, 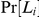, is uniform (Figure 5B). Bayes' rule provides a method for combining information about the prior distribution with the noisy neural representation, in order to compute the posterior distribution, 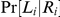 (Figure 5C). According to Bayes' rule
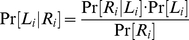
(1)where 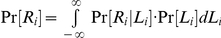.

Given a pair of neural representations, 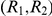, of the lengths of the first and second bars, the probability that the first bar is longer than the second bar is given by
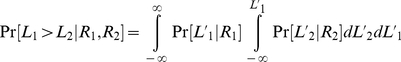
(2)This is illustrated in Figure 5D where we use a color scale to plot 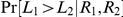 for different values of *R*_1_ and *R*_2_. The black line corresponds to values of (*R*_1_, *R*_2_) such that 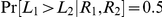. Note that the slope of this curve is smaller than 1. This results from the assumption that 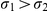, reflecting the fact that *L*_1_ has to be stored in memory, a process that may contribute additional noise to the representation of *L*_1_. An ideal Bayesian observer, who has access to *R*_1_ and *R*_2_, would report ‘*L*_1_>*L*_2_’ in trials in which 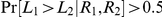 and ‘*L*_1_<*L*_2_’ in trials in which 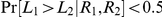. Therefore, the probability that a model would report ‘*L*_1_>*L*_2_’ in a trial in which *L*_1_ and *L*_2_ are presented is given by
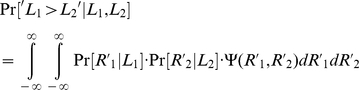
(3)where 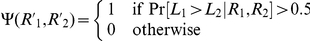.

In order to construct the response curve we compute 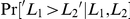 (Figure 5E). For further insights into the Bayesian computation, we consider the simple example in which the level of uncertainty in the representation of *L*_1_ is infinite, whereas there is no uncertainty in the representation of *L*_2_. In other words, 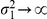 and 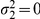. In this case, Eq. (1) becomes 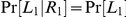 and 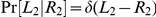, Eq. (2) becomes 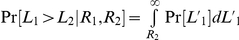 and therefore the subject would report would report ‘*L*_1_>*L*_2_’ if *R*_2_ is larger than the median of *L*_1_. In trials in which *L*_1_ = *L*_2_, Eq. (3) dictates that he would report ‘*L*_1_>*L*_2_’ in trials in which *L*_2_ is larger than the median and ‘*L*_1_<*L*_2_’ otherwise.

### Data analysis

#### Slope of line fitted to response curve

All slopes were computed after normalizing the range of lengths to 0 and 1 in the logarithmic space.

#### Bootstrap confidence intervals

We used a pairs bootstrap resampling procedure [27] in order to calculate confidence intervals for the slope of the regression lines. The bootstrap algorithm is as follows: repeated 5,000 times, we sampled (with replacement) from each subject's impossible trials in order to obtain a bootstrap dataset and fitted a regression line to the averaged response curve of each bootstrap dataset. This procedure resulted in 5,000 bootstrap slopes that could be used for calculating a CI for the slope of the regression line fitted to the experimentally obtained data points. The CIs reported in the text are 95% basic bootstrap intervals [27].

In order to compare the response curve slopes between subjects who saw 50–200 pixel lines and those who saw 150–600 pixel lines we sampled from each group independently using the algorithm above, and then constructed a 95% confidence interval on the difference between the bootstrap slopes of the two groups.

In order to compare trials with and without the interference task we calculated the difference in the bootstrap slope of each subjects' standard and interfered trials, and found the 95% confidence interval of this difference. The same method was also used to compare the slope of the response curve in the first 20 impossible trials of the experiment to the slope of the response curve in the last 20 impossible trials of the experiment.

#### Bayesian model fit

In order to compare behavioral performance to that predicted by the model, we used the model presented above to generate a set of response curves of ideal observers characterized by different values of 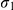 and 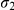. These curves were compared to the experimentally measured response curves as described below:

Note that subjects exhibited a small bias in favor of reporting ‘*L*_2_>*L*_1_’ in the 50–200 and 150–600 standard experiments. Subjects reported that ‘*L*_1_>*L*_2_’ in the impossible trials in 41% and 46% respectively. This tendency has been reported previously [28], [29]. In principle, such a bias can be explained in our Bayesian framework by claiming that the prior distribution that the subjects use in their Bayesian computation is biased in favor of small magnitudes, as was observed for speed perception [18]. In this framework, it is predicted that in the modified experiment (Figure 4A), the global bias should be larger in the trials interfered by the color task than in the standard trials. In fact we found that the global bias was larger in the modified trials (42% vs. 38%). However, this effect was not statistically significant (*p* = 0.58, two tailed *t*-test). More importantly, this explanation is circular because a bias in the opposite direction could equally well have been explained by arguing that the prior distribution is biased in favor of large magnitudes. Therefore we did not attempt to account for the global bias and subtracted it before fitting, assuming that it is generated by a different mechanism. Thus, for the purpose of finding the parameters we added a constant to each of the response curves to normalize them such that mean(Pr[‘*L*_1_>*L*_2_’]) = 0.5. For purposes of comparison, the range of the logarithm of bar lengths was normalized to lie between 0 and 1 and we used a least square fit to find the parameters that best fit the population-average experimental data. We found that the best fit model parameters for the groups who saw 50–200 and 150–600 pixel-long bars were given by 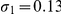, 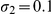; The best fits for trials not interfered by the distracting task and those that had the distracting task were 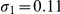, 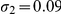, and 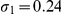, 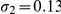, respectively.
